# Dissonance as a productive force in the emergence of alternative crisis support and impetus for social change—principles and organizational form of the association Open Dialogue Leipzig e.V.

**DOI:** 10.3389/fpsyg.2025.1426116

**Published:** 2025-02-19

**Authors:** Thomas Klatt, Lea Goncalves Crescenti, Therese Kruse, Irene Nenoff-Herchenbach, Sarah Schernau, Sebastian von Peter

**Affiliations:** ^1^Brandenburg Medical School Theodor Fontane, Brandenburg an der Havel, Germany; ^2^Offener Dialog Leipzig e.V., Leipzig, Germany

**Keywords:** Open Dialogue, crisis intervention, participatory research, learning organization, alternative psychosocial practice, grassroots, democracy, grounded theory

## Abstract

**Introduction:**

This article examines the productivity of dissonance in the development of alternative crisis intervention methods, using the German example of the “Open Dialogue Leipzige.V.” The research provides detailed insights into the development of the association and the adaptation of the OD approach to local circumstances.

**Methods:**

The presentation is based on a participatory research process, primarily processing interview data using the Grounded Theory Method. It analyzes the specific practices of implementing Open Dialogue within the association and the organizational and contextual conditions corresponding with it.

**Results:**

Despite the challenges accompanying the introduction and sustainability of Open Dialogue in the German healthcare system, the organizational structure of the association—characterized by grassroots democratic principles and a community driven by a strong willingness to change—enables a successful application of Open Dialogue principles.

**Discussion:**

The article critically illuminates how engagement, professionalization, and participatory learning mutually influence each other through the organizational form of the association, bringing forth an innovative crisis intervention that could potentially serve as a model for other contexts.

## 1 Introduction

The Open Dialogue approach is a therapeutic approach and organizational philosophy that has been developed in Finland during the 1980s. By promoting egalitarian communication by involving service users and their networks during joint processes of understanding the problems of concern and decision-making, it aims at avoiding stigmatization and to rely significantly less on medication (Olson et al., 2014; Putman, 2022a,b). The Open Dialogue approach follows 7 basic principles: (Aaltonen et al., 2011; Seikkula et al., 2011): (1) Immediate help in crises, ideally within 24 h; (2) involvement of the social network through network meetings from the beginning of the treatment; (3) flexibility and mobility with regards to the needs of the network in terms of frequency, location and participants of the network meetings; (4) responsibility for the organization and implementation of the entire treatment process by one and the same the treatment team; (5) ensuring the continuity of relationships and common understandings over the entire course of treatment; (6) tolerating uncertainty during the network meetings and (7) promoting dialogue and polyphony between network members as well as the members of staff. A more comprehensive description of the approach and of its evidence can be found elsewhere (Mosse et al., 2023).

In the German-speaking psychosocial and psychiatric care system, the implementation of the Open Dialogue (OD) approach is still in an exploratory stage also concerning its effectiveness and sustainability (Heumann et al., 2023).Thus, evidence of the effectiveness of this approach has primarily been demonstrated outside of Germany, various cohort studies providing promising results regarding clinical, economic, and social impacts (Seikkula et al., 2006; Aaltonen et al., 2011; Bergström et al., 2017, 2018). In contrast, the implementation of OD in Germany rather corresponds to a grassroots development, so far mainly driven by dedicated professional teams or individuals (Heumann et al., 2023). Among these are some bottom-up implementation approaches, which in some places also resulted from criticism of established power and care structures, a criticism that is inherent in the concept of OD (von Peter et al., 2021), often leading to challenges during implementing this approach (von Peter et al., 2022b). Thus, despite its high implementation frequency compared to the situation in international care systems, is not particularly pronounced, mainly resulting from contextual implementation barriers that widely impede the degree of implementation of OD-specific principles and features in Germany (Heumann et al., 2023).

Against this background, the question arises about alternative contextual and implementation conditions that would enable the introduction and implementation of OD in its full form in Germany and elsewhere. In this context, this manuscript focuses on a support project in Leipzig that facilitates the establishment of crisis intervention along the OD principles to a large extent. This work is part of a larger evaluation project of understanding the specific approaches to crisis intervention in the Leipzig initiative. This evaluation project was implemented in the form of two qualification theses of the first author and a student of psychology as well as collaboratively with some employees of this initiative. This manuscript presents the main results of the doctoral thesis involved and follows the research questions: (1) how did the path to implementing an alternative form of care in Leipzig unfold and how was it motivated? (2) In which ways the organizational form of this initiative corresponds with the OD approach more generally? (3) Which contextual and environmental conditions are offered by the Leipzig network that facilitate the implementation of the OD’s specific principles? Thereby, this manuscript aims at describing the mutual interrelationships between the organizational context and the specific care approach as practiced in Leipzig to illustrate the various ways in which they influence each other in creating a favorable environment to implement the OD approach.

## 2 Materials und methods

### 2.1 The initiative in Leipzig

For further insights into the approach in Leipzig and a more detailed description of crisis support as it is practiced locally, we refer you to some relevant excerpts from the association’s website in Supplementary Figure S3.

The initiative in Leipzig was founded as a formal association in 2017 with the aim of providing crisis intervention using the OD approach (Putman and Martindale, 2022). Emerging from a rather club-based and largely unfunded or minimally funded organizational structure, a challenging development process started. The beginning of this process was characterized by improvised solutions in sparsely furnished rooms, highlighting clear differences from the contextual conditions of usual professional institutions. Motivated by shared dissatisfactions with the principles and practices of conventional psychiatric care models and a strong desire for change, the group of initiators opted for OD as the central therapeutic approach. This decision led to the establishment of a suitable location and the gradual unfolding of the working practice described below.

The association first emerged from a circle of friends—a circumstance that no professional psychiatric or psychosocial service may claim as its origin. In the following years, new employees joined through contacts during the network meetings, initially working as freelancers, which in some cases evolved into permanent employment. Conversely, there are also former employees who remain connected to the association but only contribute on a freelance and occasional basis.

In the early years of the organization, new employees often started without OD training or a solid understanding of this approach. Such ‘learning by doing’ no longer occurs in this form: a thorough theoretical engagement with this approach as a minimal consensus soon after starting one’s work, followed by taking part in an established OD training, is the currently preferred path to a qualified participation in the crisis intervention program in Leipzig. In addition to outreach crisis interventions, the association also offers group support, open counseling sessions, and counseling in the sense of independent participation counseling.^1^

From the outset, peer work has played a central role in Leipzig, as also practiced elsewhere in the context of OD oriented services (Bellingham et al., 2018). Thus, a mix of various experiences are drawn upon when supporting people in crisis, involving the experiential expertise of being either a service user, and/or a family member, or a professional support worker, whereas formal-technical forms of knowledge are rather relegated to the background (von Peter et al., 2022a). In addition, people and groups of people external to the initiative in Leipzig use this context to pursue their own concerns and interests, bringing in various ideas and projects that complement the services of the main group of employees. Thus, the community of people present in the Leipzig initiative is variable: permanent and freelance employees, users, guests, interns, and researchers. In the following, all these people are summarized under the term “association.”

The work of the association does not fit into the conditions of the usual funding system for various reasons to be explained below. Thus, over the years, financial resources had to be found to finance the support work in Leipzig at least partially. The long list of sponsors includes the Health Department of the City of Leipzig, various NGOs and business support programs, foundations, and private donors. In addition, employees were organized through voluntary services, and the association collects membership fees. Currently, discussions with the psychiatry coordinator of Leipzig and the local mental health board are underway exploring possibilities of sustained funding. While the association in its early years felt little taken seriously and encountered reservations and ignorance in Leipzig, the situation seems to be changing currently. For example, a team from Leipzig University Hospital participated in OD training and subsequently worked with this approach in the context of a home treatment program. At the same time, the question is repeatedly discussed as to whether and to what extent regular funding changes the character of the service and restricts freedom in the exercise of one’s own OD practices.

The work in Leipzig is organized as a grassroot democratic form, devoting a high commitment of resources to internal communication and supervision. The ability to work productively in such team structures has become a significant criterion for employing new staff. Interactive learning of the employees has proven to be a central aspect, with both the use of competencies from previous qualifications and the discussion of the implications of this professionalization being repeatedly debated. Collaborations beyond local networks are lived out, including national and international partnerships, embedding the association in a larger context, and providing support and intellectual exchange. These aspects, as well as close collaboration within local Leipzig communities, offer promising conditions for authentically living out the principles of OD, as this work aims to demonstrate.

The employees usually document the crisis intervention work by collecting only sparingly relevant data. For the year 2020, this data was analyzed using descriptive statistics. It emerged that over this period, a total of 425 network conversations were held. Requests for crisis intervention came from networks or individuals, with users/index clients usually contacting first, followed by family members and professionals. In the period of crisis intervention considered, it was possible to involve more than one person in the dialogical meetings in about 43% of the conversations.

Further information on the association Offener Dialog Leipzig e.V. is assembled in Supplementary Figure S3.

### 2.2 The research context

The impetus for the evaluation project came from the association itself. Due to the lack of financial resources, it was decided to conduct the research as part of two qualification theses, one master’s and one doctoral thesis. The position for the doctoral thesis was advertised by a research group at the Brandenburg Medical School, which had already been involved in research on OD (von Peter et al., 2020, 2022a,b; Heumann et al., 2023).

#### 2.2.1 Research approach

Taken these collaborating partners, the project is positioned in between a collaborative (von Peter, 2017) and a community-based participatory research approach (Engage for Equity, 2023; Allweiss et al., 2024). The research team and the research members of the association were involved as partners throughout the research process, from developing the research question, through data collection and analysis, to coordinating publications. Such an approach is based on mutual learning and transparent communication, helping to align scientific investigations with the needs and priorities of the people in the research field (Unger, 2014; Wallerstein, 2018; Ackermann and Robin, 2022).

#### 2.2.2 Research participants and practice partners

This work is part of the first author’s doctoral project. Together with the last author and the master’s student of psychology, he formed the core research team (TK, JÖ, SvP), being able to contribute the most significant temporal resources for the undertaking of this research. Thereby, TK participated in all research meetings, interviews, and collaborative procedures of analysis. Additionally, he undertook a short period of participant observation, providing an excellent opportunity to deepen his understanding of the specific approach in Leipzig.

Additionally, up to five co-researchers from the Leipzig association participated in the process, contributing intensively but variably throughout the process. These individuals are as follows: LGC, TKru, IN-H, these individuals are referred to as “practice partners” (= PP) following the nomenclature of the German-speaking Network for Participative Health Research (Schaefer et al., 2022) throughout the following text.

The persons who consented to participate in the study and took part in interviews are referred to in the following as “research participants” (= RP). We contacted users of the services in Leipzig and team members, respectively, to inquire about their experiences with this support work. For reasons of data economy and following a decision in the research team, only little socio-demographic data was collected from the participants. A brief characterization of the sample can be found in Supplementary Figure S2.

#### 2.2.3 Development of study materials

During the first constitutive meeting of the research group, interesting aspects were collected, and a common thematic focus was developed. From the association’s side, there was interest in presenting and evaluating their own work with the aim of a better self-understanding and to communicate this understanding to outsiders. From the academic side, there was interest in the unique form of implementation of the OD approach and how this relates to the specific organizational form of the association. Relevant questions were collected within the team and used for constructing two interview guides (Helfferich, 2011). The interviews with the users of the service focused on their experiences and evaluations of the crisis intervention in Leipzig. During the interviews with the association’s employees, the focus was on organizational aspects. The key questions of the interviews are summarized in Table 1.

**Table 1:** Key questions of the interviews.

Interviews with service users	Interviews with team members
In which situations did you come into contact with the Leipzig Initiative?	How did you come to the association and to work in this Initiative?
What expectations did you have when you first contacted this Initiative?	How is the Open Dialogue organized in the Leipzig association?
How did you experience the support work?	How did you organize yourselves as a group and your work?
How did this support work change you situation?	What do you think is special about the Open Dialogue in Leipzig?
	How do you like your work and what do you wish for the future?

#### 2.2.4 Case selection and recruitment

Service users were asked for an interview using a contact list of the association’s network. This task was undertaken by interns of the association who were not part of the research team. In addition, a flyer was created introducing the research and distributed in various places in Leipzig. Regarding the team members to be interviewed, the PP facilitated contact with individuals who indicated a willingness to participate. Additionally, all current and former active team members were approached.

In both groups, all individuals who expressed willingness were interviewed. As indicated in Table 2, especially the team members were ready to participate. The people who declined to give an interview had various reasons for not doing so: uncertainty about the topics to be discussed; belief that they could not contribute anything relevant; discomfort in talking about sensitive topics; fear of renewed emotional stress; low confidence in research. Three interviews with users were removed from the data set. The decision was made by the team after it became clear that the work in these networks followed different procedures than Open Dialogue.

**Table 2:** Selection of participants during the recruitment process.

Group	Service users	Team members
Attempt to get into contact	76	16
Successfully contacted	57	16
Willing to participate	22	13
Excluded	3	–

There was no selection by the research team or any formalized sample principles. The search for individual cases with a special focus, in terms of theoretical sampling of Grounded Theory Methodology, occurred in the research process through two methods: first, by selection from the existing material, and second by shifting the focus of the interviews alongside the data obtained.

#### 2.2.5 Interview conduct

Using the developed interview guides, 32 semi-structured interviews were conducted with 13 team members and 22 service users, audio-recorded, and transcribed. The interviews took place in the association’s premises, at home, or in other locations, either in person or online/over the phone. They were conducted by one PP researcher and two academic researchers, with a smaller proportion of conversations with users also conducted in tandem. Conducting interviews in tandem proved to be very beneficial for collecting rich data, as the perspectives and focus of the questions complemented each other, leading to more diversity during the conversation. The transcription of most of the interviews was undertaken by research assistants and a professional service, while some were transcribed by the master’s student himself.

#### 2.2.6 Analysis

Grounded Theory Methodology was chosen for the analysis with the aim of developing a middle-range implementation theory. Qualitative data are generated and interpreted using this approach through continuous iteration of collecting, coding, and analysis to develop a theory rooted in the data. This method has been described as suitable for participatory research processes because it allows for an open and flexible approach (Charmaz, 2015).

In the project described here, a specific methodology was developed for coding the material, which can be used for both collaborative coding and individual work. This method has been extensively described elsewhere and compared with similar working methods (Klatt et al., 2025, in preparation). At this point, the coding process will be briefly described: The analysts first familiarized themselves with the material through reading or listening. Primary coding was done individually and case-specifically. For most of the material, the analysis was conducted individually, with some interviews analyzed collaboratively in tandem (one person of the research team, one PP researcher). This second working mode occurred as follows: After primary coding, the individuals involved met over several sessions to discuss and consent to categories (step a). In the second step, the focus was on 4–6 codes, which captured the essence and specificity of the text from the researchers’ perspective. This step was initially done individually (step b). Next, there was an interpersonal comparison of categories, aiming to merge similar categories and leave disparate ones. Again, the tandem selected 4–6 categories that informed the overall analysis (step c). When inserting the results into the overall project in the MAXQDA software, the consented descriptions and definitions of the codes were used to find suitable anchor examples in the source material beyond the case and connect them with the codes. This feedback served as validation of the codes and to establishing a close relationship with the primary data.

In this way, individual analysis cases were added, and the overall analysis progressed. Interim results and initial theoretical derivations were presented and discussed in research group meetings with all PP. In the final part of the analysis, theorizing was the last step of our proceeding. This theoretization of the material was undertaken by engaging with the empirical material from the interviews and various theoretical concepts that were selected to make sense of them, from both the background of OD and other theoretical fields, such as dissonance (Festinger, 2020), (de)professionalization (Grey, 2019), community of practice (Wenger-Trayner and Wenger-Trayner, 2025), translational learning (Tsimane and Downing, 2020), peer work (Bellingham et al., 2018), etc. These theoretical sources relate to the context examined in Leipzig and are the first building blocks of a ‘Grounded Theory’ of local implementation conditions. Due to the lack of resources, this theoretical work was limited in its duration, as well as its empirical grounding. Thus, further interviews or focus groups to communicatively validate the theoretical model were not possible.

#### 2.2.7 Ethical considerations

Although the participatory research approach combined with an open methodological approach sets a framework that aims for a relatively balanced relationship between researchers and participants, we are nevertheless operating in a vulnerable field in which power and dependencies play a role. Therefore, all kinds of participants were informed of their right to withdraw from the study at any time without consequences. A further requirement was that people and networks currently receiving crisis counselling were not asked to participate.

Ethical research advice and a vote were obtained from the Ethics Committee of the Brandenburg Medical School.

## 3 Results

The results presented below are a part of the developed Grounded Theory. They are divided into two main parts, which are logically connected: to answer the research questions 1 and 2, the first part presents statements from the participants (=RP) that illustrate their experiences with the conventional psychosocial/ psychiatric care systems (Section 3.1). The development of a unique way of working and organizing the support work in the Leipzig association is described in the second part as a result of these experiences (Section 3.2). An overview of these topics is given in Table 3.

**Table 3:** Themes and subthemes of the analysis.

Discomfort in relation to the system	A specific form of organization	The shape of the association’s work from the service user’s perspective
Unwelcoming care Lack of support Lack of network-perspective Painful treatment histories Illness-causing conditions	The formation of the Leipzig association Young employees at the beginning of their Professional careers Group development Networks Participation Culture of welcome Alternative culture Mutual learning	Network culture Peer involvement Alternative culture

Finally, and alongside the research question 3, the support practices in Leipzig were reviewed in relation to their fidelity to the OD principles in Section 3.3 (Olson et al., 2014). Since any implementation of fidelity principles is of little importance if not experienced by service users, this evaluation of fidelity is carried out from the users’ point of view (perceived grade of OD fidelity). Due to the length of the article, the results of this evaluation section is only presented in a tabular format.

### 3.1 Discomfort in relation to the system

Both service users and association members contributed closely related perspectives on this topic, stating that everyone is affected by the care system in some way. Individuals with an academic background reported on psychological teachings at universities (e.g., biased in scientific and treatment concepts and methods, thereby constraining ways to think differently), which cannot be listed here due to space constraints. All these topics and gaps described became the starting point for personal suffering, leading to the impulse for change by establishing the association and/or participating therein.

#### 3.1.1 Unwelcoming care

The processes and structures of the psychosocial or psychiatric treatment facilities were perceived as unwelcoming. This closeness appears as a logical consequence of the prevailing medical paradigm:

“I think such resignation also comes from the fact that, I do not know, this ward is not such an inviting place. I was there recently and was allowed in. It was like a hospital ward with neon tubes on the ceiling, a dark corridor. I would wish my sister to be able to leave there as soon as possible.” (P17N 90, service user)

At these places, service users do not feel well heard and understood. Situations of crises were classified using diagnoses, whereas different perceptions and subtle tones often go unheard or succumb to the pressure of high workloads. The clinical areas were described by the participants as characterized by hierarchies and power structures. Association members recalled their clinical experience as marked by regulations:

“I had just done an internship in child and adolescent psychiatry, and that was typical clinical routine, very hierarchical. I had a conflict because I did not address the head psychologist formally. That was a topic for several weeks, it felt like, and very structured hospital routine, many meetings, case discussions, many post-discussions within the teams only. Little contact with the patients.” (P9M 14, association member)

Further, instances of exercising power and coercion were well described.

#### 3.1.2 Lack of support

Lack of support appeared primarily to be a qualitative problem. There were rejections to support users in acute crisis, referrals, and waiting times that were not compatible with these situations. Difficulties arose when the needs of individuals go beyond what is offered:

“…you just cannot forcibly through-out someone from their apartment because they have cluttered and dirtied everything. And then send them to psychiatry, not caring at all about the state of their apartment, and then release them back into that shitty apartment, in the truest sense of this word.” (P1M 150, association member)

Social work and discharge management in clinical departments were perceived as inadequate. The life situation of the affected person beyond the clinical situation was too little considered. In some cases, hospitalization was the result of a lack of outpatient support:

“You cannot have anyone come to the house. Only the police. And that somehow does not work. So, it’s really difficult. It’s like waiting for an escalation or something, which is very terrible and which burdens everyone a lot. And, actually, this only exacerbates the whole situation and, I think, even creates it.” (P8M 73, association member)

Individuals and their networks were not well-supported during very stressful situations up to the point when it was no longer possible, and hospital treatment remained as the last option. When it came to workplace reintegration, further, service users complained about a functional orientation of assistance: those deemed unsuitable for the labor market and not dangerous to the environment gave institutions little incentive for intensive support.

#### 3.1.3 Lack of network-perspective

OD means working in networks—social and professional. In the conventional care system, the participants of our study experienced contradictory tendencies:

“…the parents are perceived to be annoying when they come and will then be sent away. There are many reservations about networks; friends are not even talked to or anything.” (P1M 162, association member)

Family members were reported to have easier access. But if the network extended beyond the usual family circle, barriers became greater:

“However, I actually went to such a family counseling center, and they said, ‘maximum two people.’” (laughs). (P3M 42, association member)

At this point, a clear difference on a paradigmatic level between widely practiced psychiatric practices and network-oriented work became apparent.

#### 3.1.4 Painful treatment histories

The painful experiences from the perspectives of the users were manifold. Often, the initial contact was already perceived as a traumatizing situation in which trust is shaken or cannot be established again:

“Yes, I would like to speak to someone,” “Yes, someone will come down soon.” That was exactly the person who called the police. And I was supposed to complain to that person […] That was quite intense. (P8N 7, user)

The way of treatment brought new problems to the users: communication failures, hospitalization and treatment with psychotropic drugs that bring side effects, application of coercion etc. Even if the treatment seemed to be ineffective, service users found it difficult to break away from it:

“Because I thought, there are also people who fight so hard to get out of there. And who still end up in the system again and again.” (P5M 86, association member)

The abundantly described painful treatment histories led to the rejection of current care and the search for alternative approaches.

#### 3.1.5 Illness-causing conditions

This approach to people in crisis was also found by the participants of our study to be largely accepted by society as normal. An excessive demand to perform weighs heavily on individuals who cannot meet these demands or fear doing so:

“You probably know the term ‘normopath’?” (P9N 13, user)

Some people cope well with this socialization and can function, others cannot. The research made it clear how deeply people were disturbed by the day-to-day pressures they experience:

“The working load, that’s why many have these diseases. Because it’s empty of meaning, the pressure is too great. What’s all this crap for, yeah?” (P9N 80, user)

The critical view of social conditions were addressed throughout the material. This critical, continually questioning attitude emerged as a commonality in both groups:

“So, a very critical and vigilant view, I think, of the classic model that exist. That’s what unites us. How is the UN-CRPD implemented? Or how are people in crisis situations, for example, dealt with? So why is it so difficult to find therapy places? Why is admission or the clinic – why is that often the answer? Why are medications often the answer?” (P10M 42, association member)

It was therefore about the relationship of the individuals in the association to the common system of psychosocial care, embedded in an overall societal system.

### 3.2 A specific form of organization

The following statements refer to the specific organizational form of the association in Leipzig. The description of the organizational form, as expected, has been derived more from the statements of the participating staff. Users primarily see the practice of crisis support, and the organizational form is not always clear to them. Accordingly, in the following sections, the quoted voices mostly stem from the association members assessed.

#### 3.2.1 The formation of the Leipzig association

The formation of the organization in Leipzig, as described above, originated from a circle of friends, which raised the question of the further development path seven years later in the history of the association: do employees still connect through the association’s work as friends?

“I sometimes have this need to involve people I know. Then I ask them if they would like to contribute as volunteers in our structures of crisis support.” (Int2M, 90, association member)

Beyond the group of people who worked more or less directly within the project, there was a veritable “scene,” in which information was circulating:

“We have a wide circle of friends, and that spread quickly in the psychiatric scene.” (Int2M, 26, association member)

The exchange of information in this circle seemed to be a difficult-to-control process.

#### 3.2.2 Young employees at the beginning of their professional careers

All employees who took part in interviews were young with an average age under 30 years. Further, during the research meetings, it became clear that there had not yet been any notable exceptions form this staffing in Leipzig. Most of the employees started working in the association immediately after completing their studies. A few had previously worked in other areas, as well within the conventional system of psychiatric care, which, however, was rather considered to be an obstacle to their job in Leipzig:

Before that, I had worked in assisted living as a caregiver. In a residential group of outpatient assisted living. In the moment, I’m an occupational therapist… But I try to forget that. Well, I kind of have to forget that to be able to work well here. Or a lot of it. (I3M, pos. 8, association member)

When the interviews talked about professionalization and its significance in the current field of work, it became apparent that the rather open constructions of professional regularly became a problem regarding financial opportunities:

With every funding application, some kinds of qualification are required by the funding institution: staff is supposed to be psychologist, or social pedagogue etc. In any case, each person must have a paper with some kind of stamp. It’s just not enough to say: “there’s someone who is in top shape to do this work.” In principle, the cat bites its tail at some point: as soon as you start saying: “can you pay us for our work?,” many people say in a friendly way: “yes, but only if you are psychologists…” Yes, but our concept says that we do not want this dependency on formal professionalization. (I7M, pos. 65, association member)

The group within the association was dynamic and open with various interfaces to the outside. Critical concerns with the conventional care systems seemed to be a crucial criterion for how the engagement of individuals was motivated (see above):

“It’s difficult to find people who see themselves affected enough to want to be involved.” (Int3M, 114, association member)

One employee appreciated the exchange within the association both among the team and with the people who seeked their help, emphasizing the positive encounters that arose from it:

“The best thing is the relationships within the team and with the people who request our help. There are so many beautiful encounters.” (Int5M, 167, association member)

The difference from the usual ways, in which teams come together was that friendships existed partially beforehand, only then followed by joint work.

#### 3.2.3 Networks

Networks could be families with experience in crises and their management. This experience was sometimes based on a long time of living together: as with other health problems, family members can become experts and bring their valuable knowledge into the work together. Employees became part of the existing social network during the support and offered relief for strenuous and long-endured situations of mutual concern:

“I once noticed that the family being there is also security. Because they also deal with these problems all the time.” (Int2M 50, association member)

In the following quote, the employee formulated a unique feature of crisis support using the OD approach: networks are the central resource for this work: the (family) system is not only the target of therapy, as in conventional approaches, but the very matrix of engagement and development to achieve change and improvement:

“The fact that we involve the networks so naturally. I think that’s already special. So not just: ‘We have to coordinate.’ but really doing crisis work in the networks.” (Int2M 114, association member)

#### 3.2.4 Participation

How did individual actions and network activities relate to each other? The employees in Leipzig tended to vary in their degree of integration into the team. Their previous life and professional experiences had an impact on their work in the association. What had been learned theoretically or from previous employment, however, at times needed to be unlearned to find one’s way into this new form of practice:

“To keep falling flat on your face and realizing, ‘Oh crap, I did it again on my own.’ does not work, in open dialogue, it does not work. You’re always in pairs.” (Int2M, 128, association member)

There were plenty of opportunities for involvement in Leipzig, the organizational structure largely based on a participatory way of working. Participation could be a welcome offer for all kind of people: for members of mental health care or private networks, other professionals, or even private acquaintances. Interested individuals arrived at the association via these ways and could have become eventually permanent employees:

“What was missing in my life was someone saying, ‘there’s a project, and you can join in.’ To condition to join is that you do an interview beforehand to speak freely and don’t omit certain statements. And then we have a project here, in which you can participate.” (Int8N, 52, user)

At the same time, there were some hurdles to such flexible participation, such as participating in training: it seemed necessary to separate crisis support and general engagement in other areas of the association. Even in other areas of the association’s work, access became somewhat more difficult in recent years due to development: the team grew and became more structured. Usually, a specific occasion was necessary for people to participate at all and to use the spaces offered continuously. This was a surprising finding considering the evidence that spoke for a successful culture of welcome in the association in Leipzig, a topic that will be thematized in the following section.

#### 3.2.5 Culture of welcome

Employees warmly welcomed users and created an open atmosphere in which they quickly felt integrated:

“And I was received as if I had always been there. The friendliness, the openness of the people who greeted me. And that was a relief for me because I’m not used to that.” (Int5N, 16, user)

Users could be themselves in the association without having to pretend anything. Emphasis was placed on authenticity, and everyone was accepted for what they are:

“You are free there and can be yourself. You do not need to pretend. You do not need to be afraid. This fear of many mentally ill people is not necessary.” (Int5N, 22, user)

The openness and inclusivity of the group were clearly recognizable. Friends, family members, and the entire social network were welcome. The association served as a safety net and offered support:

“Yes, meeting people is like nutrition, right? Encounters with people can be annoying or exhausting. But ultimately, that’s better than not having any encounters at all, right? It’s like brushing your teeth or something during those phases when things are spiraling downward, right?” (Int9N, 35, user)

The association enabled users to open and find support in a safe environment. The resulting bonds and relationships were of great importance to those involved.

#### 3.2.6 Alternative culture

Joint rejection of certain conventions brought the feeling of being in the right place:

“And that was always sympathetic, the right people who understand you. That’s such an important point when you have a psychologist from an alternative background, as when a psychologist says, ‘by the time you are in your mid-30s, is not it may be time for a wife and children? I just wasn’t in the right place for that.” (Int10N, 97, user)

“Alternative structures” with uncertain financing became familiar when individuals themselves had been activist in such structures in the past:

“I am very familiar with this system based on donations and alternative structures, it feels at home.” (Int10N, 97, user)

#### 3.2.7 Mutual learning

“There’s no strict separation: ‘we are the ones who understand, and you still have to understand it.’ I’ll put it in black and white.” (Int8N, 35, user)

Learning was a genuine dialogical practice for all participants. Individuals remained experts in their life worlds and were to be addressed as such.

External groups with their own themes and situations also became aware of this principle and used the association’s facilities and network for their services, thereby also developing the OD crisis support further:

“Over the years, people came to set up support systems for pansexuality or others. Friend circles, yes. Mutual support systems combined with house projects to jointly live-in, that sort of thing.” (Int2M, 26, association member)

The association became effective when its members knew what competencies were available and how they were distributed. Specific groups or individuals were contacted who were likely to provide good support in specific situations:

“And then I also send people into the groups, of which I know that they have experience with tapering off medications and so on.” (Int2M, 62, association member)

Several instances could be used for learning processes within the team: team meetings, intervision, supervision, team dialogue, OD training, and contact with external networks that supported or already practiced the concept. Lastly, learning took place during the support itself, during the network meetings. Two components were conducive to this process: there were always two moderators, and this practice remained consistently dialogical.

#### 3.2.8 Perceptions of the service users

The significance of networks during the crisis supports in Leipzig was frequently addressed also in interviews with the users of these support services, either as something that had shaped their relationships before accessing these services or because of this engagement. Peer involvement in networks and among employees was perceived as a difference from conventional care. Further, it became evident from the perceptions of the users that the basic principles of the OD approach could have far-reaching effects on their lives and how they experienced support during crises.

#### 3.2.9 Network culture

The interview statements provided by users shed light on various aspects of a network culture and its various influence on dealing with crises. Their perceptions on this topic were multifaceted and differentiated. Some participants simply appreciated the principle of a network culture to be a central component of OD and central to their processes of recovery.

Another participant offered a fresh definition of such a network-focus, conceptually embedding the network meetings within everyday life routines—a definition that may also reflect the special organizational features of the Leipzig association:

“I believe these networks need a new designation. Not psychotherapy, just a meeting of people, voluntarily. That’s a different matter.” (IntN11, Pos. 247, user)

Other participants underscored the inclusivity and equality they experienced within the network meetings as practiced in the Leipzig association: they felt part of the group and reported that open and unbiased dialogue with other people led to further conversations and new forms of relationships:

“How to say it? As open dialogue is so open, many people just come into contact with other people, or become aware of them, through other people. Because they have noticed that this person has also experienced something, or whatever. And then conversations arise where I never thought, where I never thought, I would eventually be able to talk to about.” (IntN5, Pos. 92, user)

After trying various solutions and their failure, also within the conventional system, users came to appreciate networking practices as a central for resolving difficult situations, by also shifting the attention away from their own problems and life situation:

“And this developed into a direction that he also wanted, that it wasn’t just about him, but that he also wanted to know how we as a group could support each other. And not just: How can we help HIM, but how can we all better relate, as a group.” (IntNP01, 13, user)

These experiences led to various learning effects that networks could also be a resource for dealing with difficult life situations, and to be actively sought again when needed.

#### 3.2.10 Peer involvement

In the context of the association in Leipzig, various aspects in relation to the perception of peer support emerged from the data. On a more general level, the widespread understanding was shared that peers share the experience of mental health problems, which was described to be supportive:

“And I generally felt understood there because I felt that the people definitely also struggle with mental crises or have had them, maybe in the past.” (IntN1, Pos. 112, user)

This led to a sense of connection and the opportunity for mutual learning, values that were also reflected in the organizational form of the Leipzig association:

“I can remember that there was someone who described their voices, and I thought to myself, wow, I’ve never heard it like that before. That was fascinating and I think that’s when I got a much better understanding of how it works with voices.” (IntN5, 78, user)

This exchange with peer workers was described to be another form of interaction oriented towards supportive exchange and mutual respect, both perceived to be fundamental to the principle of OD as well as this approach was practiced in Leipzig.

In addition, the peers were also considered “similar people,” suggesting that users did not see any categorical difference to the other employees and to themselves:

“So where I say, ‘These are similar people, they are talking to me and I’m that person.’” yes? (IntN9, 3, user)

The phrase “similar people” is theoretically interesting. It refers to closeness, which remains indeterminate, but indicates a special connection:

“I’ve actually never met people who try to fight against the psychiatric system and in general. And then I got the flyer about the open dialogue and I got to know many, many people through it. These are all people who want to go against the system. I always had the feeling that I was alone in this. And they have always tried to silence me, especially the psychiatric system.” (IntN5, item 94, user)

In this last quote, the shared feelings of dissonance appeared also in the perception of a user, also making clear that a “peer” was less understood as a support staff with lived experiences but more as a person to connect with due to shared criticism.

#### 3.2.11 Alternative culture: critical at a distance from the conventional system

Most users proved to be informed about the alternative positioning in Leipzig and the theoretical foundations underlying this form of support:

“Yes, it’s a more anti-psychiatric association that’s independent and, um, eh, yes. There is also a library with critical books. Of course, there are different ways of thinking and models. For example, I also know the socialist patients’ collective.” (IntN9, 51, user)

The association was perceived by all their users as a service outside the psychiatric care system:

“… this place is basically an anti-pole to what is understood as social normality or something like that. This requires the willingness to negotiate and to show solidarity. And that the open dialogue gives everyone the option of receiving support. This is something very special, which I very much hope that in the future there will perhaps be more such positions and more people who do such work.” (IntN15, 70, user)

The differences were seen in the type of interaction compared to those in usual therapy contexts:

“I mean, they treat everyone who comes here as they are. You are an individual. And that’s what they are there. You’re not a case number, you do not have a diagnosis.” (IntN5, 107, user)

A decisive factor here was also the political positioning of the team, which does not exist in this clarity in other support systems:

“I’ll try to describe it: suppose I had met people here who are all center- or center-right or conservative and had a very archaic idea of relationships between practitioners and patients. Or between what must happen now so, for instance, I can participate better in society, then that would not have worked. Then I would not have come back, I think, because I already have enough of that elsewhere, of such pigeonholing.” (IntN18, 56. user)

### 3.3 Perceived grade of OD fidelity

As described above, the support practices in Leipzig can only be reviewed cursorily in relation to their fidelity to the OD criteria for reasons of space. Since, as shown in the discussion, the implementation of the OD in Germany is primarily lacking in fidelity to the structural principles (Heumann et al., 2023), in the following, the degree of implementation of these principles is focused upon from the perspective of service users. Further information on this and the implementation of the therapeutic principles can be found in Table 4.

**Table 4:** Anchor citations that demonstrate the perceived fidelity to the OD principles in the Leipzig support system from the point of users.

	Key elements	Anchor citations/ quotes from the material
1	Two (or More) Therapists in the Team Meeting	“We also had conversations together… there were network dialogues with a doctor and I one or two further staff.” (IntN2, 111)
2	Participation of Family and Network	“I cannot say much about what OD means but in my memory, I think it was something like: Aha, here you are, here are four new people, one person who is sometimes not doing well and three people from her family…and then the conversation started.” (IntN17, 44)
3	Using Open-Ended Questions	“…questions, such as: “tell me, what’s going on with your day, how are you, what’s going on with you feelings”? And I think that opens-up a lot, a lot of space.” (IntN17 44, user)
4	Responding To Clients’ Utterances	“And you could also say: ‘I have to get out because of my emotions.’“, then that was okay too. Or I could sit and we did not talk at all. But just sat there. You can also have a conversation without having to say anything.” (IntN5 46, user)
5	Emphasizing the Present Moment	“I cannot pin it down to certain days as there was always something that touched me at every meeting. Emotionally, positively too. Because at that moment it was also shown that I cannot really be that crazy. That I’m just a normal person, a grown woman who has, or had, a lot of grief and worries. And not as someone sitting there who has a roof damage.” (IntN5 86, user)
6	Eliciting Multiple Viewpoints	Outer: “That was the first thing I offered, because I’m very much in favor of people knowing each other and that they can exchange information. That’s how to compare points of view.” (IntN8 25, user) Inner: “First, I had to bring it up again and make it aware and clear and then also answer questions and look at it from other angles.” (IntNP02 52, user)
7	Use of a Relational Focus in the Dialogue	Well, I think that helped us to understand each other a bit better during this crisis. I would say that the day after plus a few more hours was always quite harmonious until there was another crash somehow. But it was definitely very helpful to be able to somehow understand the other person’s perspective. (IntN16 60, user)
8	Responding to Problem Discourse or Behavior in a Matter-of-Fact Style and Attentive to Meanings	“I could talk uninhibited. That’s new me. Somehow, I was never very good at talking to the staff in the clinics. As absurd as it is, I wasn’t looked at strangely. I noticed very quickly that I could simply tell the most absurd things without noticing the reaction. So, I was taken seriously with it, it was addressed, although we were all somehow aware that it had nothing to do with reality. But still, it became real at that moment and that allowed me to open- up better.” (IntNP02 44, user)
9	Emphasizing the Clients’ Own Words and Stories, Not Symptoms	“I feel more accepted here in the state that I feel right now, and then I’m not so busy with a lot of my energy pretending or hiding something, but then I can use that energy to direct it at the real difficulties.” (IntN18 88, user)
10	Conversation Amongst Professionals (Reflections) in the Treatment Meetings	“Well, this exchange between the people who came was something special. So easy to talk about it again, to talk about it, which I sometimes felt a bit forced. Somehow because it was part of their concept. And yet I also benefited from it [laughs], I just found it a bit weird in parts.” (IntN1 80, user)
11	Being Transparent	“I also thought it was very good that it was so transparent about what is going on with the duty of confidentiality and also the inspection of documents if I want to. This gave me a lot of trust and a professionalism.” (IntN1 138, user)
12	Tolerating Uncertainty	“It’s a tightrope walk, but here it was accepted, and I wasn’t forced to lie and present myself as more stable than I was. Which, for example, I would have had to do with several therapists to be allowed to be in therapy. Because at the end of the initial consultation, they will ask you the question: ‘Can I rely on them not to do anything to themselves until our next appointment?’ And if I do not want to be taken away and if I want to have this therapy place, then I have to lie. And that’s extremely hurtful and frustrating and really does not help you seek help. This did not happen here.” (IntN18 52, user)

The *network orientation* has already been mentioned above and was strongly seen in the foreground by the users of the support services in Leipzig. *Immediate help* was common in Leipzig too. In the interviews, users were surprised by the promptness of support they received:

“… I had a crisis, and it happened very quickly; a colleague and someone else came directly and I was really able to express everything that was in me. That was really good.” (I10N63, user)

The frequent use of contemporary communication media was described contribute to this low-threshold approach:

“Modern media were used to clarify my issues relatively quickly. You do not have to reach someone on the phone during office hours, but you get an SMS or a Telegram message. This makes the whole thing easier.” (IntN18, 94, user)

Good structural solutions have been found in Leipzig also for the implementation of *flexible and continuous* support: these principles were reflected in many facets in the descriptions of the user participants of our study. The location of support and the mindset of the staff involved were largely perceived to be flexible and continuously available without too many pre-fixed schedules or assumptions. Flexibility was appreciated in the conduct of the network meetings, leading to a rather radical acceptance of the specific needs and conditions of the participants. This also applied to the principle of *responsibility:*

“Yes, definitely. I think that’s what it was all about, this taking responsibility and thinking along with you, always thinking along with a person.” (IntN16, 84, user)

Thereby, the taking-over of responsibility in Leipzig remained dynamic and was negotiated again and again during the network meetings. It was dealt with in the network, their participants assessing it together and deciding how they will distribute it. A high degree of *tolerance for uncertainty* (see citation in Table 1) supported these processes and saved energy that users often must spend on strategic behavior in relation to these questions in the psychiatric system. And finally, a high degree of fidelity to the principle *of dialogue and polyphony* played a role here:

“Support was usually at eye level, which created a very positive atmosphere for me, even if the conversations were sometimes exhausting due to their degree of negotiation. But people came together to solve a problem together.” (IntN18, 18, user)

## 4 Discussion

This manuscript presents the results of a participatory evaluation, in which the specific OD implementation practices within the context of an association in Leipzig were investigated using a Grounded Theory Methodology. During theory development, three theses emerged from the data, which will be discussed below. As mentioned above, these theses are not to be understood as a fully developed middle range theory, mainly due to a lack of resources, as described above. Thus, some terms and concepts that have emerged during the analysis were further systematized into three theses, providing for an initial theoretical frame to conceptualize the support work in Leipzig that will be further condensed in the concluding section:

1. Experiences with the mental health care system motivate committed professionals and peer support workers in Leipzig to turn away from it and to seek alternatives; these experiences facilitate the implementation of the specific form of practicing OD in Leipzig in an organizational form that currently is situated outside the system.
2. The association in Leipzig provides favorable conditions for the implementation and development of the OD approach, enabling opportunities for interactive and transformative learning that allow young professionals at the beginning of their careers to experiment; the association exhibits characteristics of a learning organization that provides fertile grounds for innovation in the field of mental health care.
3. The specific form of implementation of OD in Leipzig could serve as an example for similar processes in other environments in Germany and possibly internationally.

These theses will be discussed in the following discussion section against the broader background of implementation difficulties of the OD approach in Germany and internationally. We will address questions such as how the activities of the Leipzig network fit into this context and what organizational quality the association offers for the intended practice.

### 4.1 Cognitive dissonance as a driver for restructuring care

Discussing thesis 1, the established procedures within the conventional psychosocial or psychiatric treatment system led to cognitive dissonance (Festinger, 2020; Weinmann, 2019) among some users, committed practitioners, or laypeople, thereby laying the grounds for the alternative care practices of the Leipzig association. This concept by Festinger (1957) describes a state in which a person simultaneously holds contradictory thoughts, beliefs, or attitudes, which can lead to discomfort and often to a change of his/her attitudes or behaviors. In the context of OD, cognitive dissonance can occur when the conventional, often medicalized approach collides with wishes for alternative approaches (von Peter et al., 2022a; Skourteli et al., 2023). More closely in relation to the project in Leipzig, this dissonance served as the central link to join the network of individuals that are engaged within the context of and around this association. Closely linked to feelings of dissonance may be so-called “moral distress,” arising when professionals experience situations in which they cannot act according to their moral or ethical stances due to institutional constraints or other external factors (Kada and Lesnik, 2019; Jansen et al., 2020, 2022). In this context, our analysis seems to reveal that practicing OD may contribute to reducing this form of distress, potentially taking off some of the emotional labor related to it.

From both phenomena, thus, criticism of the prevailing paradigm of psychiatric care may arise, arguing for instance against its biomedical reductionism, and pathing, as in our project, the way for alternative forms of care that are based on more holistic approaches considering social, cultural, and psychological factors more strongly. Both the feelings of dissonance and moral distress can motivate and concretely shape alternative care practices. The project in Leipzig is not alone in this context. Other highly valuable support initiatives in Germany, in this case a user-controlled one, also emerged from strong criticism (Russo and von Peter, 2022), as well as various historical developments of support organizations in the 1970s/80s (e.g., https://www.pinel.de/, Kempker and Lehmann, 1993), pointing at the value of critical reception and attempts to overcome the discourses and practices of conventional care structures.

Against this background, at least three main approaches of dealing with the experienced shortcomings of the conventional care system can be distinguished: (1) individuals keep on suffering from the system, (2) they try to escape this distress by changing it, or (3) by focusing on efforts to develop alternatives. The latter approach has also been chosen in Leipzig, the distress and dissonance being the significant origin for the foundation of the association and the various forms of sustained engagement. This foundation marked the turn away from the conventional system of various members of the Leipzig “psychiatry-affected/critical scene,” consisting of both laypersons and dedicated professionals, embarking on a search for an alternative support culture. During this process, encounters were made with the OD approach, which seemed to offer pragmatic responses to some of this criticism. From the beginning on, a more fundamental change in the overall care system was hoped to emerge from the impetus of the rather niche existence of the association. Thus, the actors in Leipzig were not content with simply withdrawing from the system but always aimed for changes in the direction of a more comprehensive paradigm shift in the psychiatric and psychosocial care systems and related sciences (Kuhn, 1962, 2023).

On this narrow ridge, the described community in Leipzig balances with a current tendency towards increasingly anchoring itself and taking-over more responsibility within the context of the municipal psychiatric care system. Despite this trend, debates about the possibility [or theoretical impossibility (Eichinger, 2009)] to evade the criticized systems continues. Thus, criticism and resistance are dialectically interwoven with the local practices in Leipzig, productively shaping both the organizational form and the support that happens within. Related are ongoing debates on the question, by which means activist or reformist goals can or should be achieved. Concrete steps towards obtaining more secure financial resources are constantly being reflected upon also in relation to their consequences on the support currently offered and democratically voted on. In more fundamental terms, resistant groups and movements are faced with a dilemma: on the one hand, they must fear compromising their own principles and values in moving towards and with the system, while on the other hand, existential needs threaten to disappear into insignificance as an already marginal group (Burstow, 2021). Criticism and resistance must confront these ambivalences; there is no real way out.

### 4.2 The “Community of Practice” in Leipzig as favorable implementation condition for OD

To substantiate thesis 2, the concept of “Community of Practice” (CoP) served as a sensitizing concept (Lave and Wenger, 1996; Wenger-Trayner and Wenger-Trayner, 2025), defining a group of people that share a common concern or passion and gradually learn how to improve this practice together. In the context of the Leipzig association, the community supporting this practice includes not only members of the association and individuals in regular employment but also freelancers, volunteers, and service users, along with their networks and friends. This composition also recalls the older concept of a “therapeutic community” (Putman, 2022a,b), describing communitarian alliances between professional staff and services users with blurred boundaries that also emerge from shared activities and responsibilities.

Further, the notion of a therapeutic community has been further elaborated by Haigh and Pearce (2017), emphasizing core principles such as democracy, permissiveness, and communal responsibility—values that are also highly evident in the Leipzig initiative. Integrating these frameworks reveals how the Leipzig association fosters a participatory environment that encourages mutual learning and supports recovery through blurred professional-user boundaries, Further the network structure of the Leipzig community aligns with systemic thinking of the OD approach: thus, networking as one of the central principles of OD and both an organizational form and the central feature of the practices or crisis support in Leipzig intertwine in favorable ways, reinforcing each other. Thus, OD as an instrument of community building on a personal level is combined with a bottom-up-structure following grassroots democracy on the organizational level, both converging into an integrated model that focus on and aims at responding to societal concerns (Schmidt, 2017). As an alternative context of support, it holds the potential to forge connections into diverse societal spheres, possibly contributing to overall democratic developments in the larger society. In summary, the emphasis on participation, empowerment, and the activation of social networks in Leipzig has the potential to stimulate a larger cultural shift also beyond the field of psychosocial care (von Peter et al., 2022a).

Both the crises support in Leipzig and its organization involving extensive reflections and metacommunication on the jointly experienced and shaped processes, the technique of the reflecting team is central, and in Leipzig, various supervision formats are given more space compared to other health care contexts. During these formats, the participants learn from each other and support each other to integrate what they have learned back into practice—a process that can also have transformative effects on the related networks (Akinsooto et al., 2020; Tsimane and Downing, 2020). This exchange and feedback loops in all directions keeps the work in the association open to new influences and prevents the practiced OD from becoming monological or too dogmatic. The necessary listening and reaching out of the actors in the networks are fundamental features of such a learning organization (Zinner, 2014) and, further, are important for the transformation of societies and the other organizational systems developing within them.

Thus, Leipzig’s OD practices demonstrate potential for broader societal impact, aligning with Gregory’s (1982) theories of gift versus commodity exchange and social capital development. The initiative’s focus on trust-building and democratic participation exemplifies how grassroots mental health innovations can contribute to a cultural shift prioritizing collaboration and mutual empowerment over transactional relationships. This aligns with international trends emphasizing the role of social networks in promoting community well-being (Florence et al., 2020).

In this context, youth and little experience as a consistent personal characteristic of the active players in Leipzig are also striking. Youth like this makes the team more open and flexible to the path into the unknown. Further, employees are not yet socialized in conventional professional roles but are freer to look for their own professional identity. The development of the association so far has given them the space to structure their activities according to the needs of the concept and the people involved. At the same time, the process of implementing the new individuals interested to join the team requires the agile structures to succeed, a process that is hardly manageable within traditional structures, mainly basing the work on conventional competencies or working methods without too much freedom to build-out another style of support (Weber, 2014). Further, the association’s emphasis on younger professionals reflects the OD principle of “unlearning” entrenched roles and adopting a “not-knowing” stance, as described by Wilfred Bion (Simpson and French, 2001; Goddemeier, 2023). These characteristics enhance adaptability and creativity, allowing for experimental practices in a low-hierarchy setting. The youthful openness of the team facilitates the development of new professional identities unburdened by traditional psychiatric paradigms, fostering a culture of innovation and responsiveness to community needs.

Thus, the association in Leipzig can be seen as a stimulating example of a learning organization by relying only on a few structurally designed hierarchies, which benefits these learning processes. Fundamental to these processes are also the more permeable boundaries between the organization and its environment comparing them to more conventional organizational context of mental health care: both only initial steps of participation and more permanent forms of engagement are possible in Leipzig, sometimes even without a contract or without fulfilling the usual formal qualifications—a network structure that seem to function even without formalized commitments (Klärner et al., 2020). Instead, an open space has been constituted where a culture of welcome is lived, allowing people low-threshold access and exit (Carrel, 2013). Such a culture naturally raises many questions that require ongoing discussions in everyday life, also in Leipzig: How must such a space be constituted or maintained? How open must/ should it be, and who decides on its structure? In answering these questions, the participation of various people and networks external to the association in Leipzig seems to play a role too. In addition to the joint crisis support, there are a few spaces to exchange on these questions, marking smooth transitions between the status of a person that is being supported to one that may contribute to co-designing the structures of support, enabling joint creativity during this process.

In relation to these organizational peculiarities, the uniqueness of the association can be stated in two ways: firstly, in relation to the implementation of OD in Germany and internationally, and secondly, compared to other providers in the Leipzig service region.

### 4.3 OD Leipzig as a potential implementation model

The background for the third thesis is the observation that the association in Leipzig, with its very specific history and current configuration, offers a good opportunity for implementing OD in Germany. The above-reported results demonstrate that crisis support according to the principles of OD largely succeeds in Leipzig. In the results section, this fidelity was reconstructed based on the statements of users (see Table 4) that highlight the commitment to the OD key elements (Olson et al., 2014) from their perspective. Accordingly, the project offers sufficient scope on both an organizational and therapeutic level, which, compared to the OD implementations in other health care contexts in Germany, can be described as unique (Heumann et al., 2023). To illustrate this into more detail, the usual implementation problems of OD both nationally and internationally are discussed subsequently, followed by a more general elaboration on the contextual requirements for the optimal implementation of the OD approach.

As described elsewhere (Heumann et al., 2023), the implementation of OD in Germany faces various challenges at systemic, organizational, and individual levels: systemically, the fragmented healthcare system poses problems in clarifying responsibilities and necessary cooperation across sector boundaries. Further, the systemic work within networks is currently not financially rewarded. The same, usual services are oriented towards the achievement of goals and solutions as quickly as possible, with less possibilities for ongoing and potentially long-lasting network processes as practiced in the OD approach. On the organizational level, the implementation of OD often is hindered by traditional working approaches and staff turnover. It is difficult to embed the concept within an organization without obtaining sufficient support from the management level. Individually, a frequent lack of a suitable mindset among employees is described to adopt this new way of working, which at times may entail radical changes on the level of the organizational culture. In addition, the redistribution of responsibility and thus power in the therapeutic process often encounter resistance, which can lead to fatigue.

Similar problems are described internationally. Skourteli et al. (2023) summarizes findings from Scandinavia and beyond in her report before discussing challenges of OD implementation in a day clinic setting in Greece: even in Scandinavia, interprofessional cooperation is described to not always succeed, the separation by expert roles and hierarchical structures leading to uncertainties. Further, it is described how fundamental organizational change, related to the implementation of OD, provokes resistance at all levels. Outside Scandinavia, the dominant biomedical model is seen as inhibiting. From an economic perspective, the costs of training and complex accounting modalities are significant obstacles to such an implementation (Florence et al., 2020). Translating the OD approaches to local contexts and cultures, further, poses various challenges.

A central contribution of this study is the examination of Leipzig’s bottom-up OD implementation compared to top-down strategies in other contexts, such as the UK, Italy, or South Korea. Grassroots adoption in Leipzig emerged organically from the collective experiences of professionals and service users disenchanted with conventional psychiatric care. In contrast, top-down implementations often encounter challenges such as hierarchical resistance and rigid organizational structures (Skourteli et al., 2023). While top-down strategies may benefit from systematic resource allocation and training, grassroots initiatives, like Leipzig’s, capitalize on flexibility, democratic participation, and community-driven innovation. This distinction underscores the potential for hybrid models that combine grassroots dynamism with institutional support, fostering sustainable OD practices globally.

Given the current state of the mental health care system in Germany (and elsewhere?), it remains to be asked, how a change towards the direction outlined above can be fostered. For this purpose, actors within and outside the system are needed that are willing to engage disproportionately, in the sense of “engaged practitioners” (Waddoups, 2022; Bell et al., 2010), contributing to change at various levels of an organization or the wider system. In addition, theoretical contributions from research and sciences are also needed, influencing and facilitating practical developments and transformation. Methodologically, participatory and ethnographic approaches as well as action research and discourse analyses, combined with a high level of engagement (“engaged science”), appear promising for this purpose: (https://www.engagingscience.eu/en/, Keller and Limaye, 2020).

What can be learnt from our study: to implement the OD approach, organizations need flat hierarchies, transparency in their processes, and a clear orientation towards participation and collaboration. There is a need for opening the institution and for creating conditions that facilitate transformative learning. Such flattening or dissolution of existing hierarchical structures in psychiatric care institutions seems like an overwhelming task, given the current state of usual health care institutions, but this is necessary to create space for processes described above. The ideal case of a grassroots democratic structure, such as in Leipzig, despite its mainly financial shortcomings, seems to be feasible but may currently only be outside of the organizational frame of the usual care systems. Given wider developments in society (such as the dismantling of democracy, the rise of authoritarian political positions, the economization of healthcare, as well as the psychiatrization of society), always shaping the context of psychosocial work too, thus, a full implementation of OD according to all its principles, at least in Germany seems distant outside of subcultural niches (Asseburg and Goren, 2022; Zick et al., 2023; Köchert, 2015; Vaudt, 2022; von Peter et al., 2021).

On a personal level, a specific mindset seems necessary to work in line with the OD approach. Traditional expert roles must be abandoned in favor of acknowledging diverse expertise that not only draws on academic and professional sources. Working in multiprofessional teams including peer workers, and across sector boundaries should be the norm, and participants should be trained for this purpose (Hendy et al., 2023). Theoretically grounded in our data, the above-mentioned concept of a Community of Practice can be useful in this context to form a shared interest in shaping and developing support practices together. Such communities, besides their more direct engagement in OD practices, could be more involved in processes of its implementation, also to work on appropriate financing conditions on an economic level. In the association in Leipzig, for instance, individuals or groups can be found who are dedicated to political and lobbying work to drive systemic changes. A community of practice, further, could integrate research, thereby linking to staff or resources at universities, research institutes, or science shops (Benz et al., 2022).

Last, finding suitable options to account for work according to the OD principles within the healthcare system would be a recommended task for a group of “engaged economists” including health policymakers. A sustainable funding model would be a milestone towards establishing this approach in Germany. It would financially secure professional practice and allow therapists to dedicate themselves fully to this work without existential worries or without having to find ingenious structural solutions. It would be easier for management in clinical departments and other health care institutions to support OD practices and their development, providing for convincing arguments also from an economic perspective. Global treatment budgets are a new financing option for flexible and cross-setting work practices, which have already been tested for psychiatric pilot projects according to §64b SGB V (Schwarz et al., 2022). They could also promote the implementation of OD in Germany if rolled-out to a larger scale.

## 5 Conclusion

In a time when the healthcare system is increasingly being critically scrutinized, the establishment and development of a grassroot association for crisis support underscores the necessity of alternative care models. Shaped by personal experiences with the existing psychiatric system, moral distress, and cognitive dissonance, a group of individuals who had either experienced crises themselves or professionally supported others through them, leading to the aspiration to create a space for change. As described above, this manuscript is to be seen as an intermediate product on the way to a more fully developed theory. Such a development of a consistent theory in accordance with the logic of the GTM theory formation was not possible within the framework of the underlying research. Nevertheless, two theoretical derivations will be presented here in a condensed form: first. The process of dissonance reduction and second, the redistribution of power and responsibility in the mental health care system.

Concerning the process of dissonance reduction, a diagram in Supplementary Figure S4 (Supplementary Figure S4 summarizes the course of the development process of the Leipzig association. as it emerged from our research data. The straightforwardness of the diagram is a simplification of the complex processes described and for the sake of a clear visualization. Here is a summarised description of the diagram in Supplementary Figure S4): The experiences with the conventional system bring about dissonance and the search for alternatives. The common interest brings people/peers together and they look for suitable concepts. The action begins with concrete plans for implementation. The participants develop into new roles and build a learning organisation. The first results become visible: new professional identities emerge during the work experience in a new type of organisation. An innovative practice is designed, and new things are created using swarm intelligence. For the players involved, this can mean a reduction in the dissonance that triggered this process.

The fact that regression can occur again over the course of time is shown below in the topic of power and responsibility. In relation to the second conclusion, the institutions of conventional psychiatric care are powerful in multiple respects: (1) they usually operate via hierarchical structures; (2) diagnoses are used that can lead to the stigmatization of those affected; (3) coercive measures against patients can be seen as an extreme form of exercising power; and (4) the way in which funding and resources are allocated determines which treatment approaches are pursued and paid for, while others are marginalized. The path taken by the association in Leipzig shows several attempts to redistribute these forms of power. The underlying democratic impulse redistributes power to people in psycho-social emergencies, their networks and professionals that strive for support alternatives. To this end, committed practitioners are giving up positions that the traditional system holds for them in favor of working in a grassroots democratic organization that can only exist in a social niche for the time being. Economic success has yet to materialize and sustainable implementation of this support alternative is an enduring challenge.

At the same time, the results of our research suggest that this association in Leipzig, with its specific history and current structure, presents a suitable opportunity for the implementation of OD in Germany and in the local context of Leipzig. This conclusion is supported by identified organizational conditions and competencies of the involved individuals that facilitate the implementation of OD. We observe an exceptional and necessary freedom at the organizational and individual level, namely, from long-established and entrenched structures, which, compared to other implementations of the concept in Germany, can likely be described as unique.

### 5.1 Strengths, limitations, and outlook

This study demonstrates several *strengths*: the participatory approach was pursued sustainably from the outset and maintained over an extended period, fostering a thorough and inclusive process. Despite limited financial support, the research team sustained a long and continuous investigative process, marked by openness to reflection and methodological creativity. The diversity and interdisciplinarity of the research team contributed to methodological and theoretical innovations that enriched the study.

However, the study has its *limitations*: The analysis primarily relies on a retrospective view of the research object (the people interviewed had already completed their crisis support, and some staff members had not been involved in this support for some time when the interviews took part), with limited opportunities for field research in more depth. Furthermore, while dissonance and its resolution emerged as important themes, these processes could not be explored in depth at an individual level. Additionally, aspects related to the effectiveness of Open Dialogue (OD) were beyond the scope of this manuscript. In the area of theory development, further empirical steps on the path to a complete theory could not be taken. The people involved in the project were too engaged in other tasks.

Several *open questions* remain *for future research*: Can the theoretical derivations from the project be developed into a consistent middle-range theory? What steps would be necessary to follow the logic of theory formation? If the outcome of this process is promising the next step were testing the middle range implementation theory across various implementation contexts and conducting in-depth comparisons of specific historical implementation processes of OD. Further exploration of the dissonance concept and its relation to similar theories like ‘resistance’, ‘reluctance’ or ‘ambivalence’, especially within the context of professional socialization across different healthcare systems is warranted. Moreover, defining meaningful, network-related outcomes to evaluate OD effectiveness is a critical step forward, with potential comparisons to psychotherapy research focusing on systemic approaches.

## Data Availability

The datasets presented in this article are not readily available because access should be discussed in the group of participants. Requests to access the datasets should be directed to Thomas.Klatt@mhb-fontane.de.

## Footnotes

^1^ https://www.teilhabeberatung.de/
