# Supplementary material for: Dissonance as a productive force in the emergence of alternative crisis support and impetus for social change—principles and organizational form of the association Open Dialogue Leipzig e.V
Source: Front Psychol. 2025 Feb 19;16:1426116. doi: 10.3389/fpsyg.2025.1426116 (PMC11880860; doi:10.3389/fpsyg.2025.1426116)

Supplement

Figure 1

Recruitment process

group	user	Member of the association
Tried to get into contact	76	16
successfully requested	57	16
participated	22	13
excluded	3	-

Figure 2

Figure 2: Some socio-demographic aspects of the sample (interview participants):

1. average age:

o Both groups interviewed have a similar average age. The value of approx. 24,4 years (user) and 27,3 years (association member) shows that predominantly young people were interviewed in both groups.

2. experience in the psychiatric system:

o The length of experience with the conventional system was around 4 years in both groups. Only four people - exclusively users - had not yet had any contact with the system.

3. gender distribution (Diagram 1)

o Overall, slightly more women were surveyed.

o The proportion of women among employees was 90 %.

4. social/professional background: (diagrams 2 and 3)

o Users: Diagram 2 shows the occupations held by the group of users

o Employees: Diagram 3 shows the professions and qualifications encountered and their distribution among the association members surveyed

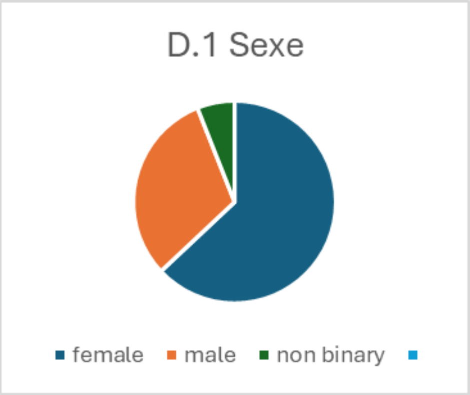

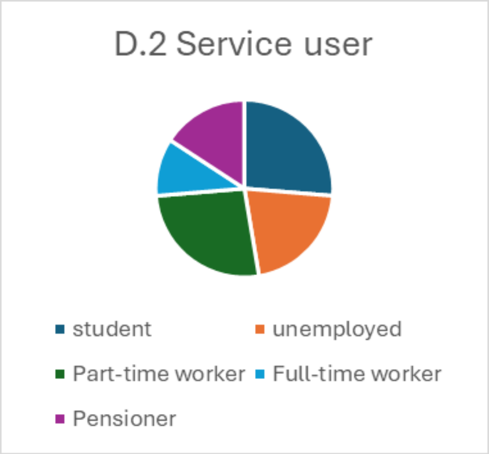

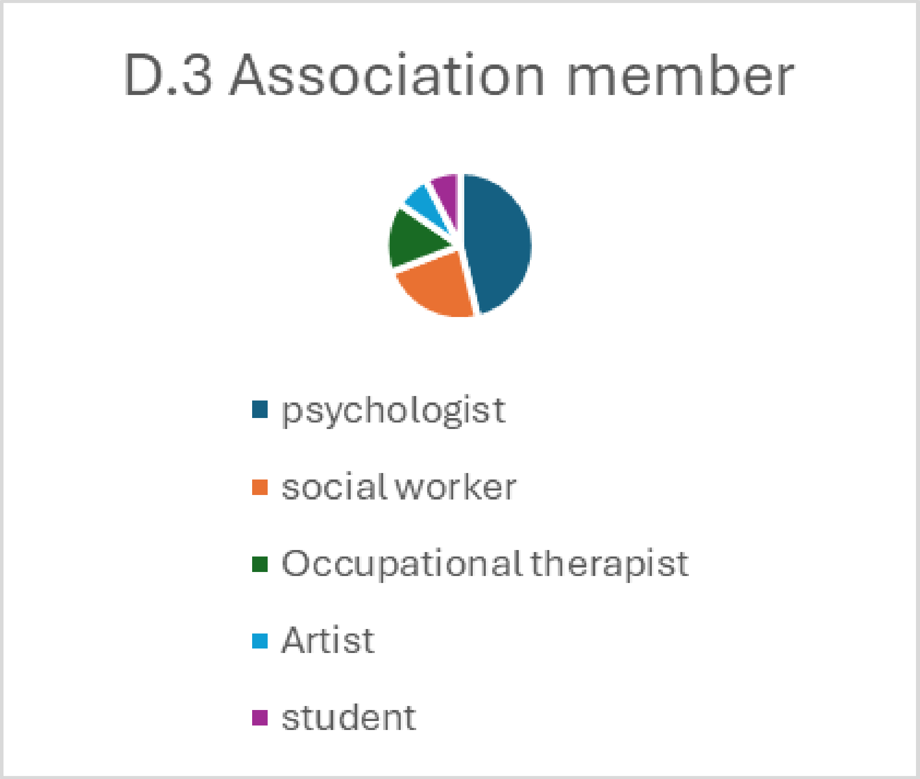

Figure 3

Excerpts from the website of the association Offener Dialog Leipzig e.V. (https://offenerdialog-ev.de/)

**Our mission statement**
We make it possible to deal with acute crises at home through open dialogue and are committed to an inclusive society through supplementary independent participation advice. It is important to us to protect the dignity and autonomy of all people. That is why we want to raise awareness of the open dialogue approach.<br>The association was founded in 2016 by a number of committed people who have made it their mission to provide an alternative way of dealing with severe psychosocial crises. In 2018, a peer counselling centre (EUTB) was also founded

to support people in dealing with barriers. You can find more information on the goals and values of the association in our pdf icon

Self-conception (short english version)

The association Offener Dialog e.V. has made it its task to support people in crises, in a way the dignity and autonomy of the people are not sold. We want to have a humane approach and an inclusive society in which everyone has their place. Because we often experience crisis as an expression and indication of problems we are all faced with. As long as violence, coercion and submission in dealing with crises are on the agenda, we have to fight for another handling with crisis. The open dialogue seems to be a good way in doing this.

Crisis support

Open dialogue is one way of dealing with acute psychosocial crises. It is based on an exchange of dialogue between everyone who is experiencing or witnessing the crisis. With this in mind, we moderate discussions between those affected, family members, friends, acquaintances, colleagues and people who can provide support.

If you need immediate help in a mental health crisis and live in Leipzig, you can contact our team. We will arrange an appointment at short notice and as quickly as possible. Our service is free of charge for you.

Through dialogue, we look for understanding and individual ways out of the crisis. Tolerance of diversity, respect for the dignity of all people and their autonomy are of particular importance to us.

Crisis support procedure

We will arrange an initial personal appointment as soon as possible - how quickly this is possible depends on our capacities. If we do not have capacity, you can contact us again a week later, when things may look completely different. Other people, such as relatives, friends and supporters, can also be invited to the meetings.

At least two of our team members will come to your home for the appointment to moderate the discussion between you and the people involved. If you do not wish this, the rooms of Offener Dialog e.V. are also available for this purpose. As a rule, a dialogue lasts 90 minutes.

What happens afterwards - whether there should be further meetings and at what intervals or what else may be needed - is decided jointly by all the people involved in the dialogue. Our team then withdraws if we are explicitly asked to do so by all parties involved or if we have jointly decided that further support from Offener Dialog e.V. is not necessary.

We work independently of diagnoses

Diagnoses can help people to defend themselves against demands or make it easier to meet people who have experienced something similar or to look for specific information.

However, we believe that it is possible to accept what is being experienced or what is currently achievable without having to justify it (e.g. through diagnoses). You can describe the phenomena you are experiencing best and much more

accurately than a diagnosis. This is because diagnoses are often accompanied by stigmatisation, helplessness and discrimination.
We want to make it possible to meet people who have had similar experiences so that they can talk about them, and we are happy to mediate.
That is why we do not give diagnoses and do not charge for diagnoses. In addition, it is not important whether you ‘have’ one (or more) diagnosis(es) so that an open dialogue can take place. We also try to create structures that make it easier for us not to think in terms of diagnoses (see Confidentiality).

Figure 4: Dissonance reduction process

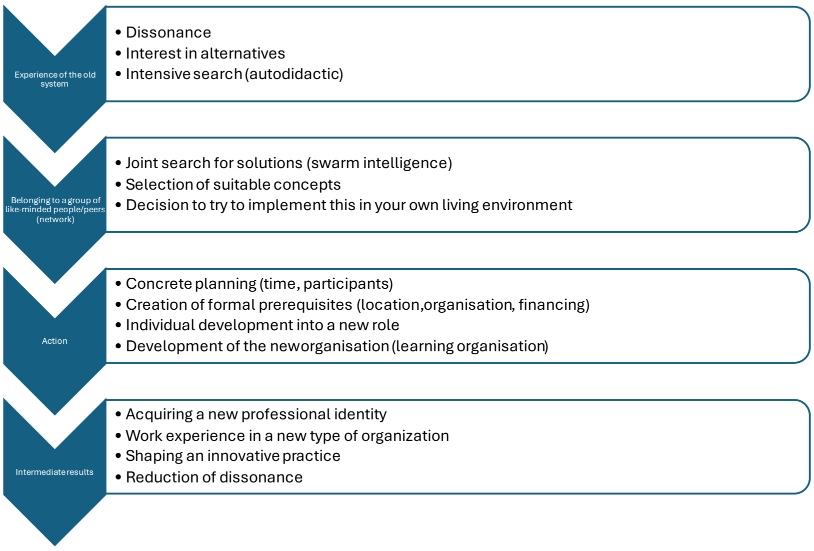

Supplement: Supplementary file 1 [file Supplementary_file_1.pdf]
